# Relationship between brain metastasis and thyroid transcription factor 1

**DOI:** 10.1038/s41598-023-29236-1

**Published:** 2023-02-02

**Authors:** Engin Kut, Serkan Menekşe

**Affiliations:** Medical Oncology Clinic of Manisa State Hospital, 45040 Manisa, Turkey

**Keywords:** Non-small-cell lung cancer, Lung cancer, Cancer, Oncology, Biomarkers, Predictive markers, Prognostic markers, Medical research, Diagnostic markers, Predictive markers

## Abstract

Brain metastases (BMs) are common in lung adenocarcinomas (ACs). Thyroid transcription factor 1 (TTF-1) is important in the diagnosis of AC. This study aimed to examine the relationship between TTF-1 and BM for the first time in literature. The data of 137 patients with AC that developed BM between 2009 and 2020 were retrospectively analyzed. A total of 137 patients, 120 (87.6%) male, and 17 (12.4%) female were examined. Their mean age was 59.78 ± 0.82 years. The Eastern Cooperative Oncology Group (ECOG) performance score was 0–1 (< 2) for 39 (28.5%) patients and 2–4 (≤ 2) for 98 (71.5%). TTF-1 was positive in 100 (73%) patients and negative in 37 (27%). More than five BMs were present in 102 (74.4%) patients and less than five in 35 (25.6%). All the patients received whole-brain radiotherapy. None of the patients was suitable for surgery or radiosurgery. The median survival time was 6.4 [95% confidence interval (CI), 5.67–7.1] months. The survival time was 7 (95% CI, 5.91–8.09) months for the TTF-1 (+) patients and 5.8 (95% CI, 4.1–7.5) months for the TTF-1 (−) patients. In the univariate analysis, there was a significant relationship between survival time and age (*p* = 0.047), TTF-1 (*p* = 0.024), and ECOG performance score (*p* = 0.002). The multivariance analysis revealed a significant relationship between survival and TTF-1 (*p* = 0.034) and ECOG score (*p* = 0.007). We found a correlation between survival time and ECOG performance score and TTF-1. TTF-1 can be used as a biomarker to monitor prognosis in the follow-up and treatment of patients with AC that develop BM.

## Introduction

Due to the advances in diagnosis and treatment in recent years, the life expectancy of cancer patients has been prolonged. However, there has been no decrease in the incidence of brain metastases (BM)^1^. Lung cancer constitutes 40–60% of BM^2–4^. The most common non-small cell lung cancer that metastasizes to the brain is lung adenocarcinoma (AC). Thyroid transcription factor 1 (TTF-1) is a transcription protein that is positive in 70–80% of ACs, and it is used in the diagnosis of AC^5^. In recent years, studies have shown that TTF-1 is not only an important diagnostic but also a prognostic and predictive marker in patients with Acs^6^. However, the relationship between BM in AC and TTF-1 remains unknown. Therefore, in this study, we retrospectively evaluated AC cases that developed BM during their follow-up in our center.

## Materials and methods

### Study population

Patients who were followed up with a diagnosis of AC and developed BM between 2009 and 2020 in the Medical Oncology Department of Manisa City Hospital were retrospectively analyzed. The study included patients with stage 4 AC, aged 18 years or older, who were found to have BM at the time of diagnosis or developed BM during their follow-up. The patients were negative for c-ros oncogene 1(ROS-1), anaplastic lymphoma kinase (ALK), and epidermal growth factor receptor (EGFR) mutation. The BM diagnosis was made based on brain magnetic resonance images.

### Data collection

The patient's demographic characteristics, such as age and sex, ECOG performance scores, number of BM, TTF-1 results, and their relationship with survival were examined. TTF-1 was examined from the first lung biopsies of the patients at the time of diagnosis in all patients Sections of 3–5 micron thickness were prepared for the immunohistochemical examination. Ventana brand fully automatic staining device (Ventana Medical System, Tucsons, AZ, USA) was used for immunohistochemical studies. TTF-1 (Clone SPT24, NovaCastra, 1:50 dilution) as primary antibody was manually instilled and incubated at 37 °C. Pneumocytes were evaluated as an internal control for TTF-1, and nuclear staining was considered positive. The patients were divided into groups according to the number of BM (< 5 and ≥ 5), the Eastern Cooperative Oncology Group (ECOG) performance score (< 2 and ≥ 2), and age (< 65, ≥ 65). To obtain the survival time of the patients, the time from the date of diagnosis to mortality or the time to the last follow-up for the patients who survived was calculated.

### Statistical analysis

Descriptive statistics were presented as mean, standard deviation, median, minimum and maximum values for numerical variables, and as numbers and percentages for categorical variables. The comparison of numerical variables between two independent groups was performed using Student’s *t*-test in case of normal distribution and the Mann–Whitney U test otherwise. Rates were compared between the groups using the chi-square analysis and Fisher’s exact test. Survival analyses were undertaken with the Kaplan–Meier method. Determinative factors were examined using the Cox regression analysis. *p* < 0.05 was considered as significant in all statistical analyses.

### Ethical approval

The study was conducted by the principles of the Declaration of Helsinki and reviewed and approved by the Health Sciences Ethics Committee of Manisa Celal Bayar University (Decision no: 20.478.486, Date 05.02.2020). This study was designed retrospectively. Informed consent has been waived by the Health Sciences Ethics Committee of Manisa Celal Bayar University.

## Results

**Figure 1:**
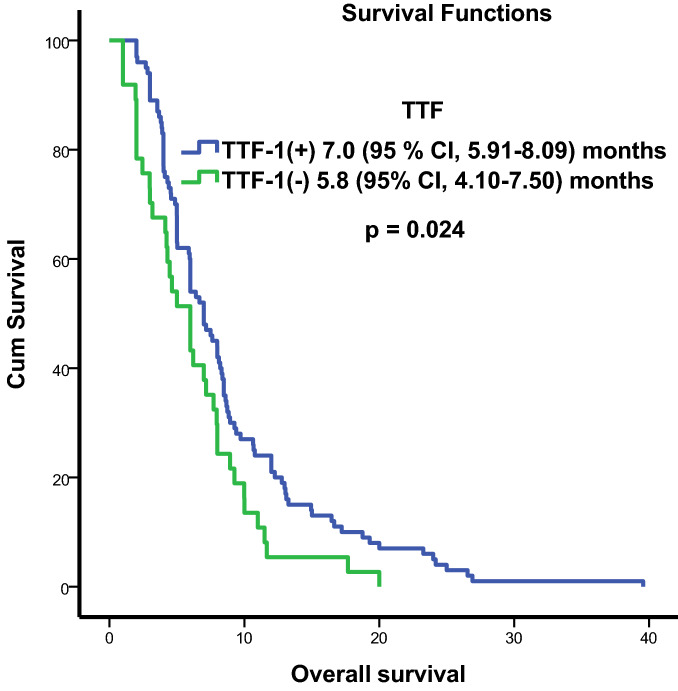
Kaplan–Meier survival curves according to Thyroid transcription factor 1 (TTF-1). The overall survival rates were significantly worse in the TTF-1 (−) group (*p* = 0.024).

**Figure 2:**
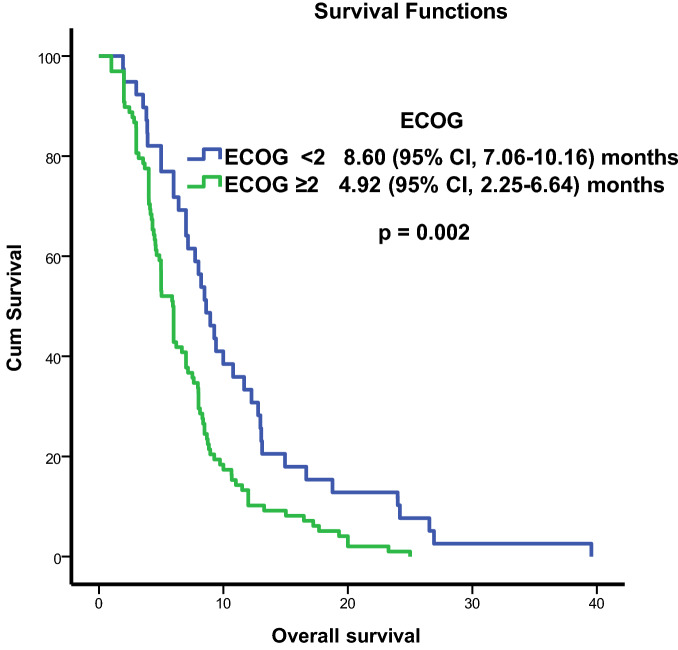
Kaplan–Meier survival curves according to Eastern Cooperative Oncology Group (ECOG) performance score. The overall survival rates were significantly worse in the ECOG performance score ≥ 2 group (*p* = 0.002).

A total of 137 patients, 120 (87.6%) male, and 17 (12.4%) female were examined. Their mean age was 59.78 ± 0.82 years. Thirty-nine (28.5%) patients had an ECOG performance score of 0–1 while 98 (71.5%) had an ECOG performance score of 2–4. TTF-1 was positive in 100 (73%) patients and negative in 37 (27%). More than five BM were present in 102 (74.4%) patients and less than five in 35 (25.6%) (Table 1). All the patients received radiotherapy to the whole brain. None of the patients was suitable for surgery or radiosurgery. The median survival time of the patients was 6.4 (95% CI, 5.67–7.1) months. The survival time was 7 (95% CI, 5.91–8.09) months for the TTF-1 (+) patients and 5.8 (95% CI, 4.1–7.5) months for the TTF-1 (−) patients. In the univariate analysis, survival time had a significant relationship with age (*p* = 0.047), TTF-1 (*p* = 0.024), and ECOG performance score (*p* = 0.002) (Figs. 1 and 2). The multivariance analysis showed a significant relationship between survival and TTF-1 (*p* = 0.034) and ECOG performance score (*p* = 0.007) (Table 2).

**Table 1:** Demographic and clinical characteristics of all patients. TTF-1, thyroid transcription factor 1; ECOG, Eastern Cooperative Oncology Group.

All			TTF-1 (+)		TTF-1 (−)		*p* value
	Number	%	Number	%	Number	%	
Sex							
Male	120	87.6	92	91	29	78	0.76
Female	17	12.4	9	9	8	22	
Age							
< 65	88	35.8	66	66	22	59.5	0.47
≥ 65	49	64.2	34	34	15	40.5	
Number of metastases							
< 5	35	25.5	22	22	13	35.1	0.11
≥ 5	102	74.5	78	78	24	64.9	
ECOG performance score							
< 2	39	28.5	27	27	12	32.4	0.53
≥ 2	98	71.5	73	73	25	67.6

**Table 2:** Correlations between overall survival and clinicopathological factors. HR, hazard ratio; CI, confidence interval; TTF-1, thyroid transcription factor 1; ECOG, Eastern Cooperative Oncology Group.

	Univariate (HR, 95% CI)	*p* value	Multivariate (HR, 95% CI)	*p* value
Age ≥ 65, < 65	1.46 (1.010–2.09)	0.047	1.03 (0.61–1.87)	0.83
Sex	1.14 (0.60–1.90)	0.60	1.06 (0.66–1.87)	0.81
Number of metastases	0.86 (0.58–1.26)	0.44	1.28 (0.91–1.89)	0.20
TTF-1	1.55 (1.06–2.28)	0.024	1.58 (1.06–2.36)	0.029
ECOG performance scor (< 2 vs. ≥ 2)	1.87 (1.20–2.70)	0.002	1.75 (1.09–2.59)	0.007

## Discussion

BM is an important cause of mortality and morbidity. With the development of BM, the life expectancy of patients is significantly shortened, reducing to four to six months. Studies have also found that the prognosis of these patients is related to the number of BM, their performance score, the presence or absence of metastasis outside the brain, and advanced age. These clinical and radiological findings are frequently used in daily practice^7–11^. However, a standard biomarker that will show the prognosis in patients with BM has not yet been found. Therefore, we conducted this study to examine the relationship between TTF-1 and BM.

TTF-1 is a transcription protein involved in the embryogenic development of the lung. It contributes to surfactant production, the development of the respiratory unit and alveoli, and normal lung functions^11,12^. TTF-1 has both oncogene and tumor suppressor properties^13^. TTF is positive in 50–60% of AC cases and it is frequently used in the diagnosis and differential diagnosis of AC^5^. It is not only a diagnostic but also a prognostic and predictive marker^6^. However, its role in patients with BM remains unknown; therefore, in the current study, for the first time in the literature, we evaluated the survival times of patients with AC with BM and their relationship with TTF-1.

In our study, the survival time of the patients was 6.4 months, consistent with previous studies. Similar to the literature, we found that patients with poor ECOG performance had a shorter survival time. We also determined that the survival time of TTF-1 (+) patients was longer, and this was statistically significant in the multivariance analysis independent of the number of BM, ECOG performance score, and age. This study is important since there is no previous research examining this relationship.

Studies examining the relationship between AC and TTF-1 have reported that TTF-1 (+) patients have a better response to chemotherapy and have longer life expectancy in both early and advanced stages^14–23^. These results have also been supported by meta-analyses^24–26^. TTF-1-positive lung cancers have a better prognosis. It is unknown why TTF-1-positive patients have a better prognosis. However, Myong et al. showed that TTF-1 expression was inversely proportional to the Ki-67 proliferation index^27^. The inverse correlation of Ki-67 with TTF expression may be one reason for the difference between TTF-1 positive and TTF-1 negatives. Although all of the patients in our study were negative for EGFR mutation, it has been reported in the literature that higher rate of EGFR mutations were detected in TTF-1 positive patients than in TTF-1 negative patients^28,29^. Patients with EGFR mutations have a longer life expectancy with the use of tyrosine kinase inhibitor drugs^29–32^. This may be another reason for the difference between TTF-1 positives and TTF-1 negatives. On the other hand, the respiratory tract is divided into the terminal respiratory unit (TRU), which plays a role in oxygen-carbon dioxide exchange, and the non-TRU, which plays a role in conducting air intake. TRU contains TTF-1-expressing cells, while non-TRU contains non-TTF-1-expressing cells^15,33^. TTF-1-positive and TTF-1-negative tumors may develop from different parts of the respiratory tract and may show different clinical courses. Another reason for the difference between TTF-1 positive and TTF-1 negative tumors may be that TTF-1 positive and negative tumors developed from different regions of the respiratory tract.

In our study, TTF-1 was evaluated from the first lung biopsies at the time of diagnosis. TTF-1 may differ between primary tumors and metastasis. But Brain metastasis is not biopsied in lung cancers because radiological findings are often sufficient in the diagnosis of brain metastases and the clinical conditions of the patients are not suitable for cranial biopsy in daily practice. This is one of the weaknesses of this study. In addition, grading data could not be obtained In this study. Because all of our cases had advanced diseases. For this reason, they were diagnosed with biopsies instead of surgical applications. Materials taken for diagnostic biopsies are usually small, so grading and differentiation are not always appropriate. Since our study was retrospective, a new biopsy could not be taken again. This is another weak point of the study.

Examination of TTF-1 only in lung biopsies in the study, and the retrospective, single-centered study with a small number of patients are the limitations of our study. However, our study is important because it is the first study to show the relationship between TTF-1 and BM.

In conclusion, TTF-1 presents as an easily obtained, inexpensive, and useful biomarker to determine prognosis in patients with AC that develop BM, when used together with the ECOG performance score.

## Data Availability

The datasets generated during and/or analyzed during the current study are available from the corresponding author upon reasonable request.
